# Safety and Immunogenicity of a DNA Vaccine With Subtype C gp120 Protein Adjuvanted With MF59 or AS01_B_: A Phase 1/2a HIV-1 Vaccine Trial

**DOI:** 10.1097/QAI.0000000000003438

**Published:** 2024-06-21

**Authors:** Nigel Garrett, One Dintwe, Cynthia L. Monaco, Megan Jones, Kelly E. Seaton, E. Chandler Church, Nicole Grunenberg, Julia Hutter, Allan deCamp, Yunda Huang, Huiyin Lu, Philipp Mann, Samuel T. Robinson, Jack Heptinstall, Ryan L. Jensen, Giuseppe Pantaleo, Song Ding, Marguerite Koutsoukos, Mina C. Hosseinipour, Olivier Van Der Meeren, Peter B. Gilbert, Guido Ferrari, Erica Andersen-Nissen, M. Juliana McElrath, Georgia D. Tomaras, Glenda E. Gray, Lawrence Corey, James G. Kublin

**Affiliations:** a Centre for the AIDS Programme of Research in South Africa (CAPRISA), University of KwaZulu-Natal, Durban, South Africa;; b Department of Public Health Medicine, School of Nursing and Public Health, University of KwaZulu-Natal, Durban, South Africa;; c Vaccine and Infectious Disease Division, Fred Hutchinson Cancer Center, Seattle, WA;; d Cape Town HVTN Immunology Laboratory, Cape Town, South Africa;; e Department of Medicine, Division of Infectious Diseases, University of Rochester Medical Center, Rochester, NY;; f Department of Microbiology and Immunology, University of Rochester Medical Center, Rochester, NY;; g Center for Human Systems Immunology, Departments of Surgery, Molecular Genetics and Microbiology, and Immunology, Duke University School of Medicine, Durham, NC;; h Division of AIDS, National Institute of Allergy and Infectious Diseases, National Institutes of Health, Bethesda, MD;; i Division of Immunology and Allergy, Centre Hospitalier Universitaire Vaudois, University of Lausanne, Lausanne, Switzerland;; j EuroVacc Foundation, Lausanne, Switzerland;; k GlaxoSmithKline, Wavre, Belgium;; l University of North Carolina at Chapel Hill, Chapel Hill, NC;; m UNC Project-Malawi, Lilongwe, Malawi;; n GlaxoSmithKline, Rixensart, Belgium; and; o South African Medical Research Council, Tygerberg, South Africa.

**Keywords:** HIV, vaccine, DNA, prime-boost, co-administration, adjuvant, MF59, AS01B

## Abstract

**Background:**

An effective vaccine is required to end the HIV pandemic. We evaluated the safety and immunogenicity of a DNA (DNA-HIV-PT123) vaccine with low- or high-dose bivalent (TV1.C and 1086.C glycoprotein 120) subtype C envelope protein combinations, adjuvanted with MF59 or AS01_B_.

**Methods:**

HIV Vaccine Trials Network (HVTN)108 was a randomized, placebo-controlled, double-blind, phase 1/2a trial conducted in the United States and South Africa. HIV-negative adults were randomly assigned to 1 of 7 intervention arms or placebo to assess DNA prime with DNA/protein/adjuvant boosts, DNA/protein/adjuvant co-administration, and low-dose protein/adjuvant regimens. HVTN111 trial participants who received an identical regimen were also included. Outcomes included safety and immunogenicity 2 weeks and 6 months after final vaccination.

**Results:**

From June 2016 to July 2018, 400 participants were enrolled (N = 334 HVTN108, N = 66 HVTN111); 370 received vaccine and 30 received placebo. There were 48 grade 3 and 3 grade 4 reactogenicity events among 39/400 (9.8%) participants, and 32 mild/moderate-related adverse events in 23/400 (5.8%) participants. All intervention groups demonstrated high IgG response rates (>89%) and high magnitudes to HIV-1 Env gp120 and gp140 proteins; response rates for AS01_B_-adjuvanted groups approached 100%. V1V2 IgG magnitude, Fc-mediated functions, IgG3 Env response rates, and CD4^+^ T-cell response magnitudes and rates were higher in the AS01_B_-adjuvanted groups. The AS01_B_-adjuvanted low-dose protein elicited greater IgG responses than the higher protein dose.

**Conclusions:**

The vaccine regimens were generally well tolerated. Co-administration of DNA with AS01_B_-adjuvanted bivalent Env gp120 elicited the strongest humoral responses; AS01_B_-adjuvanted regimens elicited stronger CD4^+^ T-cell responses, justifying further evaluation.

ClinicalTrials.gov registration: NCT02915016, registered 26 September 2016.

## Abstract 2

Supplemental Digital Content is Available in the Text.

## INTRODUCTION

Despite progress in HIV prevention and treatment, an estimated 1.3 million people were newly infected with HIV in 2022,^1^ highlighting the urgent need for an effective vaccine. To date, the RV144 trial remains the only HIV vaccine trial that has demonstrated partial efficacy against acquisition.^2^ The Pox-Protein Public-Private Partnership (P5) was established with the aim of improving RV144 by developing a vaccine capable of protecting against a broader diversity of HIV strains and achieving a better understanding of immune responses associated with preventing HIV infection.^3^ Vaccine concepts in the P5 program have focused on clade C immunogens, targeting predominant strains of East and Southern Africa, where approximately half of the 39 million people living with HIV reside.^1^

The RV144 regimen, originally designed to protect against subtype B/E strains, was adapted to incorporate clade C antigens and adjuvanted with MF59.^4^ This regimen demonstrated adequate immunogenicity in the HIV Vaccine Trials Network (HVTN)100 phase 1/2a trial^5^ and was further evaluated in the HVTN702 efficacy trial in South Africa, but ultimately discontinued due to nonefficacy.^6^ In parallel, the P5 designed the correlates program: a series of phase 1/2a trials to evaluate vaccine candidates based on favorable immune profiles of putative correlates of protection. These trials employed novel prime-boost and co-administration regimens, varied protein doses, and used new adjuvants and vaccine delivery systems, with an emphasis on shared immunological endpoints to allow for cross-study comparisons.

Preclinical studies have shown promising immune responses using DNA/protein combination vaccines.^7,8^ A comparison of responses between HVTN100 (canarypox viral vector (ALVAC)) and HVTN111 (DNA) trials indicated that DNA priming with a protein boost led to increased antibody and cellular responses compared with priming with the canarypox vector.^9^ In the HVTN105 trial, both a DNA prime-protein boost and a co-administration regimen induced potent and durable V1/V2 binding antibody responses (a known correlate of lower HIV-1 infection risk in RV144), with co-administration inducing early antibody responses.^10^ Furthermore, in the HVTN096 trial, including gp120 Env protein at the priming stage, co-administered with either vaccinia virus vaccine vector (NYVAC) or DNA, elicited earlier and even greater antibody responses.^11^

The adjuvant system 01 (AS01) has been successfully tested in vaccine trials for other infectious diseases including malaria,^12^ shingles,^13,14^ and tuberculosis.^15^ Some HIV vaccine studies have also used AS01 and have shown that it contributes to the induction of robust and persistent cellular and humoral responses.^16,17^ MF59 has likewise been used in several licensed vaccines and preclinical studies,^18^ inducing strong and durable T-cell memory and humoral responses. MF59 was also used in HVTN studies with ALVAC^5^ and was therefore chosen for comparison with AS01_B_ in this trial.

Thus, the aim of the HVTN108 trial was to evaluate the safety and immunogenicity of the DNA vaccine with different HIV clade C protein doses, adjuvanted with MF59 or AS01_B_, and dosed in prime-boost or co-administration regimens.

## METHODS

### Study Design

**FIGURE 1.**
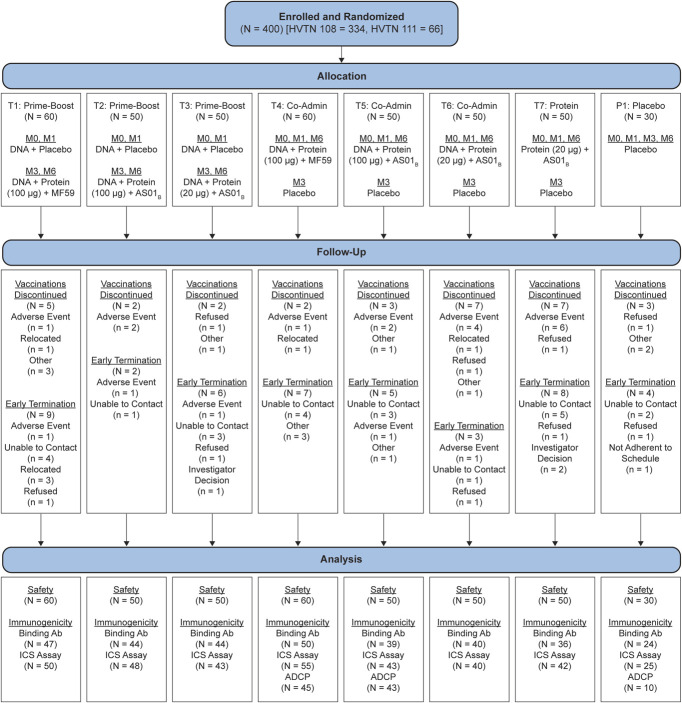
HVTN 108 (with 66 included HVTN 111 participants) CONSORT diagram. Enrollment and follow-up of participants in HVTN 108 and selected participants in HVTN 111, including availability of samples for immunologic testing. Overlap may exist between “Vaccination Discontinued” and “Early Termination” participants, as those lost to follow-up during vaccination could fall into both categories. Ab, antibody; ICS, intracellular cytokine staining; M, month.

HVTN108 was a multicenter, phase 1/2a, randomized, double-blind, placebo-controlled trial. We randomly allocated participants to 1 of 7 treatment groups or placebo at 17 clinical research sites in the United States and South Africa. Vaccinations were administered at enrollment and Months 1, 3, and 6. Participants were followed for 12 months. Vaccine regimens included DNA priming at enrollment and Month 1 with DNA/protein/adjuvant boosts at Months 3 and 6; DNA/protein/adjuvant co-administration at enrollment and Months 1 and 6; and low-dose protein/AS01_B_ alone at enrollment and Months 1 and 6 (Fig. 1, see Table 1, Supplemental Digital Content, http://links.lww.com/QAI/C284). Safety was assessed by a collection of reactogenicity and adverse events (AEs). Humoral and cellular responses were measured 2 weeks (peak, Month 6.5) and 6 months (durability, Month 12) after the Month 6 injection. HVTN111 was a randomized, double-blind, placebo-controlled trial in Zambian, Tanzanian, and South African sites comparing the safety and immunogenicity of DNA prime followed by DNA/protein boost with DNA/protein co-administration injected intramuscularly through either needle/syringe or biojector 2 weeks after the final (Month 6) vaccination.^19^ As prespecified in the study protocol, data from 66 HIV-negative adult HVTN111 participants who received identical regimens to one of the HVTN108 treatment or placebo groups were included in the immunogenicity analysis.

### Participants

HVTN108 enrolled 334 HIV-negative adults aged 18–40 years of good general health. Participants were assessed as having a low likelihood of HIV acquisition, agreed to all study requirements, and provided written informed consent. Good general health was determined by medical history, physical examination, and laboratory tests. All participants assigned female sex at birth agreed to consistent use of contraception; pregnant or breastfeeding persons were excluded (full eligibility criteria in Table 2, Supplemental Digital Content, http://links.lww.com/QAI/C284). HVTN108 and HVTN111 were approved by research ethics committees of participating sites and were registered with ClinicalTrials.gov (NCT02915016 and NCT02997969) and the South African National Clinical Trials Registry (DOH-27-1015-5117 and DOH-27-0715-4947).

### Study Products

The DNA vaccine, DNA-HIV-PT123 (IPPOX Foundation), comprised a mixture of 3 DNA plasmids in a 1:1:1 ratio, each at 1.33 mg/mL (total 4 mg): subtype C ZM96 *gag*, subtype C ZM96 *gp140*, and subtype C CN54 *pol-nef*. The bivalent subtype C gp120 Env protein vaccine comprised subtype C TV1.C gp120 Env and subtype C 1086.C gp120 Env (GSK, Rixensart, Belgium), each at either 20 μg (low dose) or 100 μg (high dose). The protein vaccine was mixed with MF59 adjuvant (Seqirus, Parkville, Australia) or AS01_B_ adjuvant, a GSK Adjuvant System (Agenus, Lexington, MA) and liposome. The placebo was 0.9% sodium chloride.

### Randomization

Randomization was determined by computer-generated sequences provided to sites through a web-based system and performed in blocks to ensure balance across groups and was stratified by geographical region. At each institution, a designated pharmacist was responsible for dispensing study products and maintaining the security of the product assignments. Participants and other site staff were blinded to group assignments.

### Safety Assessments

Participants were followed for 12 months after the initial vaccination, with safety evaluations and procedures per the schedule in the study protocol. AEs were reported over 30 days after each vaccination visit, and a subset of AEs, including AEs of special interests (AESIs) and serious AEs, were reported throughout this study.

### Laboratory Procedures

The immunogenicity objectives were to determine differences in vaccine-induced immune responses between prime-boost and co-administration regimens, regimens adjuvanted with AS01_B_ or MF59, and regimens using low- or high-dose protein. All laboratory assays (described below) were performed blinded to the treatment group with validated or qualified methods assessing peak immunogenicity (Month 6.5) and durability (Month 12). Specific antigens used in immunogenicity assays are presented in Table 3, Supplemental Digital Content, http://links.lww.com/QAI/C284.

#### Binding Antibody Multiplex Assay

HIV-1-specific IgG and IgG3 binding antibody responses were measured by Binding Antibody Multiplex assay, as described previously,^9,19–24^ at a 1:50 dilution. Tested antigens and assay reagents included vaccine-matched subtype C 96ZM651.C gp140, V1V2 antigens 1086.C V1V2 and CaseA2_gp70_V1V2.B, and heterologous antigen to assess breadth (Clade A. 00MSA gp140). All assays were conducted according to Good Clinical Laboratory Practice guidelines, including tracking of controls with Levey-Jennings charts.

#### ADCP and ADCC

The ability of vaccine-induced antibodies to engage Fc receptors and mediate antibody-dependent cellular phagocytosis (ADCP) by monocytes was measured as previously described.^25,26^ A phagocytic score was determined based on the ratio of the experimental sample to the no-antibody control. The mean phagocytosis score was calculated as follows: (% bead positive for participant × mean fluorescence intensity bead positive for participant)/(% bead positive for the no-antibody control × mean fluorescence intensity bead positive for the no-antibody control). Samples were run in duplicate within each assay and the average scores of the replicates were reported.

GranToxiLux antibody-dependent cell-mediated cytotoxicity and the antibody-dependent cellular cytotoxicity (ADCC)-Luc assays were performed as previously described.^27,28^ Additional details are provided in the Methods, Supplemental Digital Content, http://links.lww.com/QAI/C284.

### Intracellular Cytokine Staining Assay

Peripheral blood mononuclear cells, collected at peak and durability immunogenicity time points, were isolated and cryopreserved from whole blood, as previously described.^29^ T-cell responses to vaccine-matched antigens (ENV ZM96.C gp140, 1086.C gp120, and TV1.C gp120) were measured by intracellular cytokine staining as previously described^30,31^ (Materials and Table 4, Supplemental Digital Content, http://links.lww.com/QAI/C284).

### Statistical Analysis

Safety data were analyzed regardless of how many vaccinations participants received. Study enrollment was simultaneous with the first vaccination, thus all participants received at least 1 vaccination and provided safety data. Participants who discontinued vaccination were encouraged to remain in this study for safety follow-up.

Immune responses were summarized by the proportion of participants with a positive response to individual antigens at each time point, with boxplots showing the distributions of the immune response magnitudes among positive responders. Barnard exact and Wilcoxon^32^ rank sum tests were used to compare the response rates and magnitudes for responders, respectively, between the 2 groups. Two-sided 95% confidence intervals for binomial proportions were calculated using the Wilson score method.^33^ All tests were two-sided with no adjustment for multiple comparisons; differences were considered statistically significant at *P* < 0.05. SAS (version 9.4; SAS Institute, Cary, NC) and R statistical software (version 4.0.4; R Foundation for Statistical Computing, Vienna, Austria) were used for statistical analysis.

## RESULTS

A total of 400 participants were enrolled at 20 United States and African clinical research sites between 23 June 2016 and 25 July 2018. The median age at enrollment was 25 years (interquartile range 22–28), 214 (53.5%) were assigned female sex at birth, and participants had diverse racial backgrounds (Table 1). Vaccinations were completed in 369 (92.3%) participants, and 343 (85.8%) reached study completion (Fig. 1).

**TABLE 1.** Baseline Demographics of Participants in the HVTN 108 and HVTN 111 Trials

	T1 P-B, 100 µg, MF59 (n = 60: 30 HVTN 108, 30 HVTN 111) (%)	T2 P-B, 100 µg, AS01_B_ (n = 50: All HVTN 108) (%)	T3 P-B, 20 µg, AS01_B_ (n = 50: All HVTN 108) (%)	T4 C-A, 100 µg, MF59 (n = 60: 30 HVTN 108, 30 HVTN 111) (%)	T5 C-A, 100 µg, AS01_B_ (n = 50: All HVTN 108) (%)	T6 C-A, 20 µg, AS01_B_ (n = 50: All HVTN 108) (%)	T7 Ptn, 20 µg, AS01_B_ (n = 50: All HVTN 108) (%)	P1 Placebo (n = 30: 24 HVTN 108, 6 HVTN 111) (%)	Total (N = 400) (%)
Sex									
Male	23 (38.3)	28 (56.0)	26 (52.0)	22 (36.7)	20 (40.0)	25 (50.0)	28 (56.0)	14 (46.7)	186 (46.5)
Female	37 (61.7)	22 (44.0)	24 (48.0)	38 (63.3)	30 (60.0)	25 (50.0)	22 (44.0)	16 (53.3)	214 (53.5)
Race									
Black	37 (61.7)	26 (52.0)	29 (58.0)	39 (65.0)	26 (52.0)	23 (46.0)	27 (54.0)	17 (56.7)	224 (56.0)
White	18 (30.0)	19 (38.0)	16 (32.0)	17 (28.3)	18 (36.0)	17 (34.0)	16 (32.0)	13 (43.3)	134 (33.5)
Asian	4 (6.7)	2 (4.0)	2 (4.0)	2 (3.3)	4 (8.0)	4 (8.0)	2 (4.0)	0 (0)	20 (5.0)
Multiracial	1 (1.7)	3 (6.0)	0 (0)	2 (3.3)	1 (2.0)	2 (4.0)	2 (4.0)	0 (0)	11 (2.8)
Native American/Alaskan Native	0 (0)	0 (0)	0 (0)	0 (0)	1 (2.0)	0 (0)	0 (0)	0 (0)	1 (0.3)
Other	0 (0)	0 (0)	3 (6.0)	0 (0)	0 (0)	4 (8.0)	3 (6.0)	0 (0)	10 (2.5)
Age (yrs)									
Median (IQR)	24 (21–26.5)	25 (21–28)	25 (22–29)	24 (21–27.5)	27 (22–30)	25 (21–29)	25 (22–28)	26 (22–29)	25 (22–28)
18–20	13 (21.7)	9 (18.0)	5 (10.0)	12 (20.0)	6 (12.0)	9 (18.0)	7 (14.0)	3 (10.0)	64 (16.0)
21–30	39 (65.0)	35 (70.0)	39 (78.0)	42 (70.0)	32 (64.0)	33 (66.0)	36 (72.0)	21 (70.0)	277 (69.3)
31–40	8 (13.3)	6 (12.0)	6 (12.0)	6 (10.0)	12 (24.0)	8 (16.0)	7 (14.0)	6 (20.0)	59 (14.8)
Country									
United States	26 (43.3)	27 (54.0)	27 (54.0)	26 (43.3)	27 (54.0)	27 (54.0)	27 (54.0)	15 (50.0)	202 (50.5)
South Africa	21 (35.0)	23 (46.0)	23 (46.0)	21 (35.0)	23 (46.0)	23 (46.0)	23 (46.0)	13 (43.3)	170 (42.5)
Tanzania	6 (10.0)	0 (0)	0 (0)	7 (11.7)	0 (0)	0 (0)	0 (0)	1 (3.3)	14 (3.5)
Zambia	7 (11.7)	0 (0)	0 (0)	6 (10.0)	0 (0)	0 (0)	0 (0)	1 (3.3)	14 (3.5)

C-A, co-administration; P-B, prime-boost; Ptn, protein.

**FIGURE 2.**
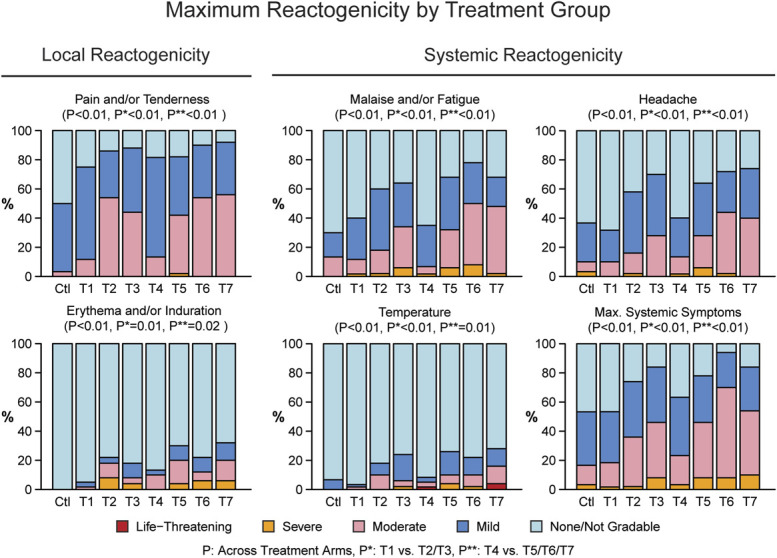
Maximum local and systemic reactogenicity events. Bar graphs show the percentage of participants in each treatment group reporting each reactogenicity event. Comparisons are made across treatment arms (P), between adjuvants in prime-boost regimens (P*), and between adjuvants in co-administration and protein-only regimens (P**). There were no grade 4 local reactogenicity complications.

There were 48 grade 3 and 3 grade 4 reactogenicity events among 39/400 (9.8%) participants. Of the grade 3 events, there were 22 local and 26 systemic events. All local reactogenicity events occurred in the deltoid region where the protein and adjuvant were injected. There were 14 grade 3 erythema events, 7 grade 3 induration events, and 1 grade 3 tenderness event. Of note, all grade 3 local reactogenicity events were in participants that received the AS01_B_ adjuvant (Fig. 2, see Table 5, Supplemental Digital Content, http://links.lww.com/QAI/C284). Of the 3 grade 4 events (all fevers), 2 occurred in group T7 (low-dose protein/AS01_B_ co-administration), and 1 occurred in group T4 (DNA/protein/MF59 co-administration). All fevers were self-limiting and resolved within 1 day. Twelve participants (3.0%) discontinued vaccinations due to reactogenicity events (7 due to erythema and/or induration, 2 due to fever, and 3 due to systemic reactogenicity events). Eleven of these received AS01_B_.

An additional 32 product-related AEs were reported in 23/400 (5.8%) participants. These included 28 grade 1 AEs and 4 grade 2 AEs, 87.5% of which started within 4 days of vaccination (see Table 6, Supplemental Digital Content, http://links.lww.com/QAI/C284). No AEs of special interests or related serious AEs were reported. There were no clinically significant differences in AEs between treatment groups.

**FIGURE 3.**
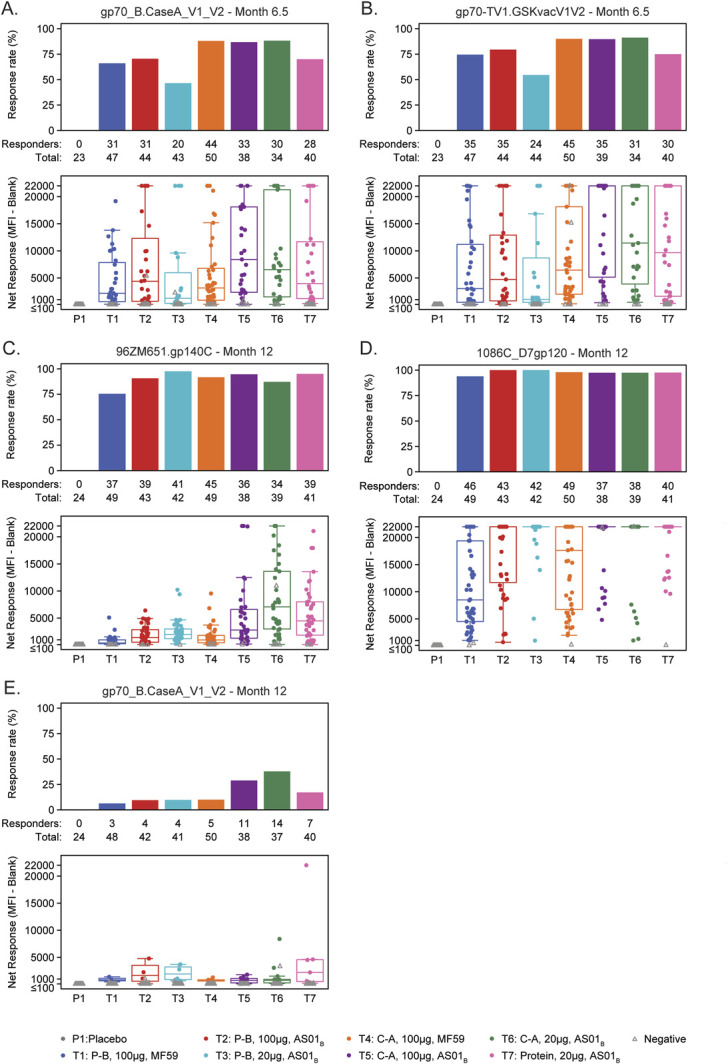
Antibody responses 2 weeks (Month 6.5) and 6 months (Month 12) after completion of the primary vaccine regimen. IgG response rate (bar charts) and magnitude (boxplots) 2 weeks or 6 months after the final immunization by treatment arm for various antigens. C-A, co-administration; P-B, prime-boost.

HIV-1-specific IgG serum binding antibody responses in all intervention groups showed high response rates and magnitudes to clade C, vaccine-matched HIV-1 Env gp120 and gp140 proteins and heterologous proteins at Month 6.5 (94.4%–100% response rates) (see Figure 1, Supplemental Digital Content, http://links.lww.com/QAI/C284). Responses at Month 6.5 to scaffolded V1V2, including the gp70_B.CaseA_V1_V2 and vaccine-matched gp70−TV1.GSKvacV1V2 antigens, ranged from 46.5% to 91.2% (Figs. 3A, B). Response rates and magnitudes remained high at the Month 12 (durability) timepoint, particularly to the vaccine-matched 1086C gp120 protein (Figs. 3C–E).

Median antibody response magnitudes among positive responders for Env IgG binding were higher in AS01_B_-adjuvanted groups for both prime-boost (T1 vs T2) and co-administration regimens (T4 vs T5) compared with MF59-adjuvanted groups at Month 6.5 and Month 12 (Figs. 3A–E). Among all participants, at Month 6.5, the median response magnitudes were higher in the AS01_B_ vs MF59 co-administration groups to gp70_B.CaseA_V1_V2 (*P* = 0.041). At Month 12, response magnitudes were higher in the AS01_B_ vs MF59 co-administration groups to gp70_B.CaseA_V1_V2, 96ZM651 gp140, and 1086C gp120 proteins (all *P* < 0.001, Figs. 3C–E, see Figure 2B, Supplemental Digital Content, http://links.lww.com/QAI/C284). At Month 12, the highest clade C gp140 responses were observed in the low-dose protein/AS01_B_ co-administration group (T6), with a higher magnitude response than the high-dose AS01_B_ co-administration group (T5) (96ZM651.gp140C, *P* = 0.023).

IgG3 responses to gp120 Env, gp140 Env, and V1V2 proteins were observed in all vaccine groups at Month 6.5 (see Figures 3A, C, E, Supplemental Digital Content, http://links.lww.com/QAI/C284). Response rates were generally lower to gp140 Env antigens and V1V2 panel antigens. For all gp120, gp140, and V1V2 proteins, the IgG3 responses were generally lower at Month 12 compared with Month 6.5 (see Figures 3B, D, F, Supplemental Digital Content, http://links.lww.com/QAI/C284).

**FIGURE 4.**
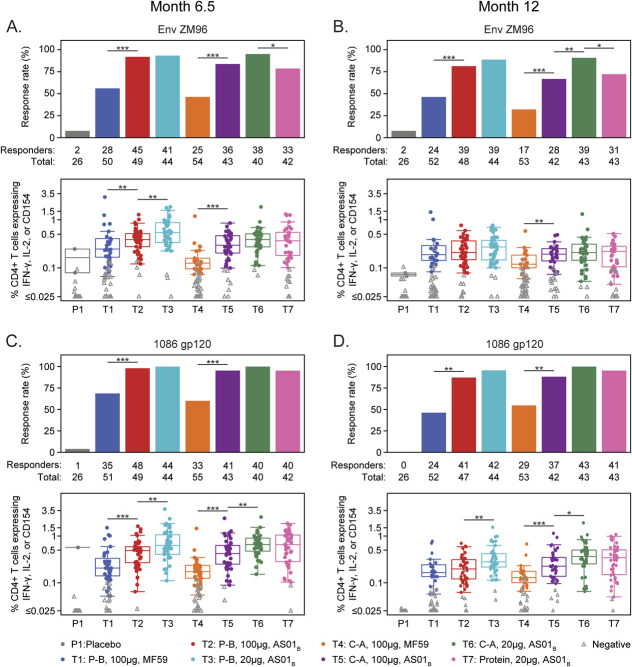
CD4^+^ T-cell responses, as measured by ICS. The CD4^+^ T-cell response rates (bar charts) and magnitude (boxplots) 2 weeks after (Month 6.5) and 6 (Month 12) months after the final immunization by treatment arm for the following vaccine-matched antigens: A, B, Env-ZM96 (where Env-ZM96 is the sum of Env-1-ZM96 and Env-2-ZM6 peptide pool responses) and C, D, 1086 gp120. Bar charts show positive response rates. Boxplots show responses and are based on positive responders only (shown as colored circles), negative responders are shown as gray triangles. *P < 0.05, **P < 0.01, ***P < 0.001. C-A, co-administration; ICS, intracellular cytokine staining; P-B, prime-boost.

ADCP activity among participants was increased at Month 6.5 in the AS01_B_-adjuvanted co-administration group (T5) compared with the MF59-adjuvanted co-administration group (T4) (see Figure 4A, Supplemental Digital Content, http://links.lww.com/QAI/C284). Similarly, for ADCC functionality, response rates were significantly higher in the AS01_B_- vs the MF59-adjuvanted group at Month 6.5, as were response magnitudes among all participants based on the luciferase assay with cells infected with either vaccine-matched infectious molecular clones (see Figure 4B, Supplemental Digital Content, http://links.lww.com/QAI/C284). Of note, by contrast to what was observed with infected target cells, rates and magnitudes of ADCC responses at Month 6.5 did not differ between AS01_B_- or MF59-adjuvanted groups when the gp120-coated target cells were used as targets, indicating a selective effect on epitope-specific functions by the adjuvants.

T-cell responses to the vector insert, Env-ZM96.C gp140, and to the vaccine-matched protein, 1086.C gp120, were evaluated, specifically comparing responses between the same regimens adjuvanted with MF59 or AS01_B_. HIV-specific CD4^+^ T cells expressing IFN-γ and/or IL-2 and/or CD40L were induced in most vaccine recipients at all timepoints and in all treatment groups. In the prime-boost high-dose protein regimen groups (T1 vs T2), CD4^+^ T-cell response rates to Env-ZM96 gp140 were higher at Month 6.5 and 12 in the AS01_B_- vs MF59-adjuvanted group (*P* < 0.001 and *P* = 0.0003, respectively); response magnitudes among positive responders were higher in the AS01_B_-adjuvanted groups at Month 6.5 (*P* = 0.021), but comparable at Month 12 (Figs. 4A, B). The 1086.C gp120-specific CD4^+^ T-cell response rates were higher in the AS01_B_-adjuvanted group at Month 6.5 and 12 (*P* = 0.0001 and *P* < 0.001, respectively), while the response magnitude was only higher in the AS01_B_-adjuvanted group at Month 6.5 (*P* = 0.001), but not at Month 12 (Figs. 4C, D).

In the co-administration high-dose groups (T4 vs T5), the CD4^+^ T-cell response rate to Env-ZM96 gp140 was higher in the AS01_B_- vs MF59-adjuvanted group Month 6.5 and Month 12 (*P* = 0.0002 and *P* = 0.0008); the response magnitude was also higher at both timepoints (*P* < 0.001 and *P* = 0.028) (Figs. 4A, B). The 1086.C gp120-specific CD4^+^ T-cell response rates and magnitudes in the co-administration groups were both higher in AS01_B_- vs MF59-adjuvanted group at Months 6.5 and 12 (*P* < 0.05, Figs. 4C, D).

In the AS01_B_-adjuvanted prime-boost regimens (T2 vs T3), there were no differences in the Env-ZM96 gp140 specific and 1086.C gp120-specific CD4^+^ T-cell response rates at Month 6.5 and 12 between the low- and high-dose protein. By contrast, the Env-ZM96-specific CD4^+^ T-cell response magnitudes were higher at Month 6.5 (*P* = 0.008), and the 1086 gp120-specific CD4^+^ T-cell response magnitudes were higher at Months 6.5 and 12 in the low- vs high-dose protein groups (*P* = 0.005 and *P* = 0.002, respectively).

In the AS01_B_-adjuvanted co-administration groups (T5 vs T6), the ENV-ZM96 gp140- and 1086 gp120-specific CD4^+^ T-cell response rates were higher in the low-dose protein groups at Month 12 (*P* = 0.0075 and *P* = 0.0205, respectively). There were no significant differences in the Env-ZM96-specific CD4^+^ T-cell response magnitudes, but the 1086.C gp120-specific CD4^+^ T-cell response magnitudes were higher in the low-dose vs high-dose protein group at Months 6.5 and 12 (*P* = 0.003 and *P* = 0.025).

Of note, the Env-ZM96 gp140- and 1086 gp120-specific CD4^+^ T-cell response rates and magnitudes in the low-dose protein co-administration regimen (AS01_B_ adjuvant) did not show significant differences compared with the low-dose protein/adjuvant only regimen (T3 vs T6, Fig. 4).

Overall, there were no significant changes in the CD4^+^ T-cell response rates to all antigens between Month 6.5 and Month 12, but the response magnitudes decreased from Month 6.5 to Month 12 in all treatment groups regardless of adjuvant, protein dose, or regimen (*P* < 0.001, see Figure 2A, Supplemental Digital Content, http://links.lww.com/QAI/C284). Of note, while statistical comparisons were not done, the same regimen induced comparable CD4^+^ T-cell response rates and magnitudes whether administered as prime-boost or co-administered. Very few CD8^+^ T-cell responses were induced across groups (see Figure 5, Supplemental Digital Content, http://links.lww.com/QAI/C284).

## DISCUSSION

In this phase 1/2a HIV vaccine trial, we assessed a DNA/protein vaccine with varying dosage regimens, protein doses, and adjuvants. All study groups had acceptable safety profiles, although more reactogenicity events were reported in the AS01_B_-adjuvanted groups. Overall, all vaccine groups showed high IgG response rates and magnitude to gp120 and gp140, and moderate-to-high response rates and magnitude to Env V1V2. The AS01_B_-adjuvanted DNA/protein co-administration regimen induced more durable antibody responses than the other regimens and showed higher phagocytosis scores than the MF59-adjuvanted co-administration regimen. The AS01_B_-adjuvanted regimens induced higher CD4^+^ T-cell responses that persisted even 6 months after the last vaccination. Furthermore, we found that prime-boost or co-administration regimens including the lower protein dose induced immune responses comparable with or better than those induced with the higher dose. As humoral and cellular responses were strong, and antibodies were more durable, the co-administration regimen merits further evaluation.

DNA, proteins, and combinations thereof, with or without other constructs have been previously evaluated as immunogens in HIV vaccines.^9–11^ However, these studies included a variety of vaccine candidates in different combinations, doses, and injection schedules. Furthermore, immunological assessments were performed in a variety of different laboratories using different assays. HVTN108, as part of P5, was optimized to compare regimens directly.

HVTN108 aimed to characterize immune responses elicited by regimens containing DNA and adjuvanted protein without a poxviral vector to down-select vaccine candidates for efficacy testing. In preclinical models, co-administration of DNA/protein elicited more robust humoral immunity than DNA alone or a prime-boost strategy.^7,8^ In humans, using a DNA prime-gp140 protein boost regimen yielded high levels of Env-binding antibodies and homologous neutralizing antibodies compared with protein alone, as well as robust and highly polyfunctional CD4^+^ T-cell responses to Env antigens. In HVTN111, DNA prime followed by DNA/protein boost was compared with DNA/protein co-administration; the co-administration regimen was associated with an increased HIV-1 V1/V2 antibody response rate, a known correlate of decreased HIV-1 infection risk in RV144.^19^

We showed that DNA prime alone followed by DNA and protein/adjuvant boosts elicited robust Env-specific CD4^+^ T-cell, antibody, and ADCP/ADCC responses. While co-administration of DNA and protein/adjuvant induced comparable CD4^+^ T-cell responses to the prime-boost regimens, binding antibody responses were considerably higher and more durable. Of interest, the AS01_B_-adjuvanted 20 µg protein administered alone induced comparable humoral and cellular response rates to the 20 µg DNA/protein co-administration regimen. These results highlight the potential potency of protein-based vaccines combined with an immunogenic adjuvant, but further assessment of the quality of immune responses is required to understand the potential impact of this regimen.

Adjuvants enhance the quality and durability of vaccine-induced immune responses. The MF59 adjuvant is used in flu vaccines due to its ability to improve antibody affinity maturation, targeted epitope breadth, and binding affinity and to elicit balanced Th1/Th2 responses.^34^ AS01_B_ has been used in several non-HIV vaccine candidates because of its ability to enhance the induction of durable immune responses.^12,35,36^ The superior CD4^+^ T-cell induction associated with AS01_B_ supports this adjuvant system for further HIV vaccine evaluation. Consistent with previous studies, we have shown that both cellular and humoral responses were significantly higher in the AS01_B_-adjuvanted groups and that these responses were durable. Given that IgG3 response rates were also increased with AS01_B_ and that IgG3 is associated with improved ADCP function,^25^ AS01_B_ likely influenced the Fc region of antibodies resulting in modified interactions with cellular Fc receptors.

We assessed whether a high-dose protein co-administered with AS01_B_ may overstimulate the immune system and thereby dampen or suppress responses. In HVTN041, a combination vaccine (NefTat and gp120W61D) formulated with AS02A was administered with varying doses (5, 20, and 100 µg) of gp120 protein. While participants developed durable gp120-specific binding antibodies, a dampening effect on CD4^+^ T-cell responses occurred at the highest dose.^37^ We evaluated similar regimens containing either 20 or 100 µg of the bivalent Env proteins adjuvanted with AS01_B_ and found similar results for cellular and humoral responses.

Strengths of our study included the randomized controlled trial design with a relatively large sample size, including diverse participants recruited on 2 continents, thereby increasing generalizability. We also compared multiple vaccine strategies with different schedules, adjuvants, and components in the same trial. A limitation of prime-boost and co-administration group comparisons was that co-administration participants received 3 protein doses while prime-boost participants received 2. Furthermore, HVTN108 was a phase 1/2a trial, meaning that it did not include HIV infection endpoints, so extrapolating whether the elicited immune responses in the promising regimens translate into protection remains unknown and would require a larger trial. Similar to previous HIV vaccine trials,^19^ few CD8^+^ T-cell responses were elicited by any of the regimens in this trial, indicating that new approaches may be needed to activate this important line of defense.

While the induction of broadly neutralizing antibodies is a priority in HIV vaccine design, evidence is building for the role of Fc effector functions and nonneutralizing antibodies in HIV prevention. For example, enhancement of Fc effector functions of broadly neutralizing antibodies is being explored for passive immunization strategies.^38–40^ IgG3 antibodies have demonstrated enhanced effector functions, including ADCC and ADCP due to the longer hinge region compared with other IgG subclasses. This study provided insights into the elicitation of IgG and IgG3 antibodies and effector functions in regimens utilizing MF59 and AS01_B_, in addition to the varied dosing regimens. Analyses are ongoing to determine potential correlations between observed responses and to compare these data to those of the HVTN702 trial, where significant correlations between humoral and cellular responses and HIV-1 acquisition were observed.^41^

HVTN108 showed that prime-boost and DNA/protein/adjuvant co-administration vaccination strategies were generally well tolerated. Combination administration of DNA/protein/AS01_B_ elicited the strongest humoral responses and AS01_B_-adjuvanted regimens elicited stronger CD4^+^ T-cell responses and antibody functions compared with MF59, providing important new insights into these vaccine products and suggesting that they may be valuable components of vaccine regimens evaluated in future trials.

## Supplementary Material
