# Supplementary material for: Safety and Immunogenicity of a DNA Vaccine With Subtype C gp120 Protein Adjuvanted With MF59 or AS01B: A Phase 1/2a HIV-1 Vaccine Trial
Source: J Acquir Immune Defic Syndr. 2024 Jun 21;96(4):350–60. doi: 10.1097/QAI.0000000000003438 (PMC11195930; doi:10.1097/QAI.0000000000003438)
Supplement: Supplementary file 1 [file qai-96-350-s001.docx]

**Supplementary Materials**

**Supplementary Methods** 1

**Supplementary Table 1.** HVTN 108 Trial Schema including HVTN 111 participants 3

**Supplementary Table 2.** Eligibility Criteria for HVTN 108 trial enrolment 3

**Supplementary Table 3.** Details of the BAMA, ICS, ADCP and ADCC antigens, including HIV-1 viral strain information 5

**Supplementary Table 4.** Panel of antibodies used for Intracellular cytokine staining (ICS) 6

**Supplementary Table 5.** Severe (Grade 3) and potentially life-threatening (Grade 4) reactogenicity events 6

**Supplementary Table 6.** Related adverse events 7

**Supplementary Figure 1.** IgG response rates (bar charts) and magnitude (boxplots) against 96ZM651.gp140C (A) and 1086C_D7gp120 (B) two weeks after the final immunization (Month 6.5) by treatment arm 8

**Supplementary Figure 2.** Cellular responses 2 weeks and 6 months after completion of the primary vaccine regimen. Response rate (bar charts) and magnitude (boxplots) by treatment arm for the following

vaccine-matched peptide pools: (A) CD4 T cell responses to Env-ZM96 gp140 and 1086 gp120 (B) IgG

responses to 1086 gp120 and 96ZM. Bar charts show positive response rates. Boxplots show responses and are based on positive responders only (shown as colored circles), negative responders are shown as grey triangles 9

**Supplementary Figure 3.** IgG3 responses 2 weeks (Month 6.5) and 6 months (Month 6.5) after

completion of the primary vaccine regimen. IgG3 response rate (bar charts) and magnitude (boxplots)

after the final immunization by treatment arm for the following antigens: 1086 gp120 (A and B), 96ZM

gp140 (C and D), and gp70 B.CaseA V1V2 (E and F), 2 weeks and 6 months after the final vaccination.

(P-B = Prime-Boost, C-A = Co-Administration). 10

**Supplementary Figure 4.** Antibody-dependent cellular phagocytosis and cellular cytotoxicity 2 weeks

after completion of the vaccine regimen. Antibody-dependent cellular phagocytosis scores to 1086 gp140 (A)

and antibody-dependent cellular cytotoxicity scores to 1086C (B). Bar charts show positive response rates.

Boxplots show responses and are based on positive responders only (shown as colored circles), negative

responders are shown as grey triangles. *P<0.05, **P<0.01, ***P<0.001. (C-A = Co-Administration). 11

**Supplementary Figure 5.** The CD8+ T-cell responses 2 weeks (month 6.5) and 6 months (month 12) after completion of the primary vaccine regimen. Response rate (bar charts) and magnitude (boxplots) two weeks after and 6 months after the final immunization by treatment arm for the following vaccine-matched antigens (A and B) Any Env (the highest response to the Env peptide pools) (C and D) 1086 gp120. Bar charts show positive response rates. Boxplots show responses and are based on positive responders only (shown as colored circles), negative responders are shown as grey triangles 12

**Supplementary Material References** 13

**Supplementary Methods**

**Supplementary humoral and cellular assay information**

Two different qualified assays were used to evaluate the magnitude of ADCC responses at peak immunogenicity. The GranToxiLux ADCC assay, performed as previously described,^1^ used the CEM.NKRCCR5 CD4+ T cell line as source of target cells by coating with recombinant gp120s representing subtype C HIV-1 vaccine-matched envelopes (TV1 and 1086).^2^ The second assay was a modified version of a previously published ADCC luciferase procedure ^1^

ADCC was quantified as net percent granzyme B (GzB) activity, defined as the percentage of target cells positive for GTL (an indicator of GzB uptake) minus the percentage of target cells positive for GTL incubated with effector cells alone (no-antibody control). The positivity criteria were ≥8% GzB activity. For the assaying using CEM.NKRCCR5 cells^2^ as targets for ADCC luciferase assays after infection by one of the two subtype C HIV-1 vaccine-matched IMCs 1086.c and TV-1 (**Supplementary Table 3**), a response was defined as positive if the peak baseline-subtracted % loss luciferase activity was ≥ 10% for either the 1:50 or 1:200 dilution.

**Intracellular cytokine staining (ICS) to measure antigen-specific CD4+ T-cell responses**

Peripheral blood mononuclear cells, collected at month 6,5, 12, 12,5 and 18 timepoint were isolated and cryopreserved from whole blood as previously described.^3^ T cell response to Env.ZM96.C gp140, Env1086.C gp120, Env TV1.C gp120 and Gag-ZM96 were measured using the intracellular cytokine staining assay as previously described ^4^ using a 17-color ICS panel.^5^ Briefly, cryopreserved PBMC were thawed, incubated overnight and stimulated on day 2 for six hours at 37°C with either peptide pools (peptides of 15 amino acids overlapping in sequence by 11 amino acids) , dimethyl sulfoxide (DMSO, 0.5%, Sigma Aldrich; negative control) or staphylococcal enterotoxin B (SEB, 0.25 µg/mL; Sigma Aldrich; positive control) in the presence of costimulatory antibodies CD28 and CD49d (1 µg/ml, BD Biosciences) and brefeldin A (BFA, 10 µg/ml, Sigma Aldrich). Cells were incubated with ethylenediaminetetraacetic acid (EDTA, 2 mM, Life Technologies) overnight at 4°C, then stained with a 17-color antibody staining panel and acquired on a BD Fortessa flow cytometer (BD Biosciences) and data were analyzed using FlowJo version 9.9.4 (FlowJo LLC).

For a given antigen, marker, and T-cell-subset, the “magnitude” of the immune response are measured by the log10 net percent of antigen-specific cells in the T cell subset that express the marker, comparing antigen-stimulated cells vs. unstimulated samples. Net percentages less than 0.01 are set equal to 0.01 (1/10,000). The following data were excluded from analysis: unreliable samples, visits outside allowable visit window, or the mean negative control responses for IFN-γ/IL-2 > 0.1%. Records were excluded if the number of CD4+ T-cell subsets or the number of CD8+ T-cell subsets was <5,000.

The magnitude of response reported, or “net response,” is the difference between the stimulated and the average of the two unstimulated wells of the percent of CD4+ T cells that express at least one of the markers in the subset: IL-2 or IFN-γ or CD40L. This percent was calculated as the sum of the cell counts across all 3 Boolean combinations of the markers divided by the total number of CD4+ T cells.

The positive response definition described below was applied to the aggregate data for IL-2 and IFN-γ and the filtering for high background or low CD4+ cell count (described in the main methods section for Env-specific CD4+ T cells) was applied to these aggregate data also.

Binary response variables (i.e., “positive”/ “negative” responses) were defined as follows. To assess positivity for a peptide pool within a T-cell subset, a two-by-two contingency table was constructed comparing the HIV-1 peptide stimulated and negative control data. The four entries in each table are the number of cells positive for IFN-$\gamma$ or IL-2 or CD40L and the number of cells negative for IFN-$\gamma$ or IL-2 or CD40L, for both the stimulated and the negative control data. If both negative control replicates are included, then the sum of the number of total cells and the sum of the number of positive cells was used. A one-sided Fisher’s exact test was applied to the table, testing whether the number of cytokine-producing cells for the stimulated data is equal to that for the negative control data. Since multiple individual tests (for each peptide pool) were conducted simultaneously, a multiplicity adjustment was made to the individual peptide pool p-values using the Bonferroni-Holm adjustment method. If the adjusted p-value for a peptide pool was $\leq$ 0.00001, the response to the peptide pool for the T-cell subset was considered positive. Because the sample sizes (i.e., total cell counts for the T-cell subset) was large, e.g., as high as 100,000 cells, the Fisher’s exact test has high power to reject the null hypothesis for very small differences. Therefore, the adjusted p-value significance threshold was chosen stringently ($\leq$ 0.00001). If at least one peptide pool for a specific HIV-1 protein was positive, then the overall response to the protein was considered positive. If any peptide pool was positive for a T-cell subset, then the overall response for that T-cell subset was considered positive.

**Supplementary Table 1.** HVTN 108 Trial Schema including HVTN 111 participants

| **Group** | **N (HVTN 108)** | **N (HVTN 111)** | | **Dose of   each protein** | **Deltoid** | **Month 0   (Day 0)** | **Month 1   (Day 28)** | **Month 3   (Day 84)** | **Month 6   (Day 168)** |
| --- | --- | --- | --- | --- | --- | --- | --- | --- | --- |
| T1 | 30 | 30 | 100 µg | | Left | DNA | DNA | DNA | DNA |
|  |  |  |  |  | Right | Placebo | Placebo | Protein + MF59 | Protein + MF59 |
| T2 | 50 | 0 | 100 µg | | Left | DNA | DNA | DNA | DNA |
|  |  |  |  |  | Right | Placebo | Placebo | Protein + AS01_B_ | Protein + AS01_B_ |
| T3 | 50 | 0 | 20 µg | | Left | DNA | DNA | DNA | DNA |
|  |  |  |  |  | Right | Placebo | Placebo | Protein + AS01_B_ | Protein + AS01_B_ |
| T4 | 30 | 30 | 100 µg | | Left | DNA | DNA | Placebo | DNA |
|  |  |  |  |  | Right | Protein + MF59 | Protein + MF59 | Placebo | Protein + MF59 |
| T5 | 50 | 0 | 100 µg | | Left | DNA | DNA | Placebo | DNA |
|  |  |  |  |  | Right | Protein + AS01_B_ | Protein + AS01_B_ | Placebo | Protein + AS01_B_ |
| T6 | 50 | 0 | 20 µg | | Left | DNA | DNA | Placebo | DNA |
|  |  |  |  |  | Right | Protein + AS01_B_ | Protein + AS01_B_ | Placebo | Protein + AS01_B_ |
| T7 | 50 | 0 | 20 µg | | Left | Placebo | Placebo | Placebo | Placebo |
|  |  |  |  |  | Right | Protein + AS01_B_ | Protein + AS01_B_ | Placebo | Protein + AS01_B_ |
| P1 | 24 | 6 |  | | Left | Placebo | Placebo | Placebo | Placebo |
|  |  |  |  |  | Right | Placebo | Placebo | Placebo | Placebo |
| Total | 400 (370 vaccinees; 30 placebo) | | | | | | | | |

**Supplementary Table 2.** Eligibility Criteria for HVTN 108 trial enrolment

| **Inclusion Criteria:** |
| --- |
| Age 18-40 years |
| Willing and able to consent, understand study concepts, and attend all visits |
| Good general health |
| Assessed by clinic staff as “low risk” for HIV infection |
| Haemoglobin ≥ 11.0 g/dL for participants assigned female at birth, ≥ 13.0 g/dL for participants assigned male at birth |
| Total white blood cell (WBC) count between 3,300 and 12,000 cells/mm^3^ |
| Total lymphocyte count ≥ 800 cells/mm^3^ |
| Remaining differential either within institutional normal range or with site physician approval |
| Platelet Count between 125,000 and 550,000 mcL |
| Alanine transaminase, aspartate transaminase and alkaline phosphatase < 1.25 times the institutional upper limit of normal; creatinine ≤ institutional upper limit of normal. |
| Negative for HIV 1 and 2, Hepatitis B surface antigen, and Hepatitis C antibody |
| Negative urine glucose, negative or trace urine protein, and negative or trace urine haemoglobin |
| Persons of reproductive potential (assigned female at birth, has not undergone total hysterectomy or bilateral oophorectomy) must have a negative serum or urine pregnancy test on day of initial vaccination |
| Persons of reproductive potential in the USA must agree to use effective contraception from 21 days prior to enrolment through the final study visit. Accepted forms include:  Condoms (male or female) with or without a spermicide, Diaphragm or cervical cap with spermicide, IUD, Hormonal contraception, or Successful vasectomy in the male partner (considered successful if a volunteer reports that a male partner has documentation of azoospermia by microscopy, or a vasectomy more than 2 years ago with no resultant pregnancy despite sexual activity post vasectomy), Sexual abstinence |
| Persons of reproductive potential in South Africa must agree to use effective contraception from 21 days prior to enrolment through the final study visit. Accepted forms include:  Condoms (male or female)  Diaphragm or cervical cap  PLUS 1 of the following methods:  Intrauterine device (IUD), Hormonal contraception (in accordance with applicable national contraception guidelines), or Successful vasectomy in the male partner (considered successful if a volunteer reports that a male partner has documentation of azoospermia by microscopy, or a vasectomy more than 2 years ago with no resultant pregnancy despite sexual activity post vasectomy), or Sexual abstinence |
| Participants assigned female at birth must have a Pap smear reported as normal or ASCUS within the past 3 years OR a Pap smear reported as normal or ASCUS AND negative high-risk HPV testing within the past 5 years. |
| **Exclusion Criteria:** |
| Blood products received within 120 days of initial vaccination |
| Any investigational agents received within 30 days of initial vaccination |
| BMI ≥ 40; or BMI ≥ 35 with 2 or more of the following: systolic blood pressure > 140 mm Hg, diastolic blood pressure > 90 mm Hg, current smoker, known hyperlipidaemia |
| Intent to participate in another interventional research study during the duration of the study |
| Pregnancy or breastfeeding |
| Active duty or reserve US military |
| Received prior HIV vaccine product as part of a study (if known to have received placebo/control, will determine on case-by-case basis) |
| Live attenuated vaccine (other than influenza) received within 30 days prior to first vaccine dose or planned within 14 days after receiving first vaccine |
| Influenza vaccine or any vaccine that is not live attenuated received within 14 days prior to first vaccine dose |
| Any allergy treatment with antigen injection within 30 days prior to first vaccine or 14 days after first vaccine |
| Receipt of immunosuppressive medications within 168 days prior to first vaccination  (Not exclusionary: [1] corticosteroid nasal spray; [2] inhaled corticosteroids; [3] topical corticosteroids for mild, uncomplicated dermatitis; or [4] a single course of oral/parenteral corticosteroids at doses < 2 mg/kg/day and length of therapy < 11 days with completion at least 30 days prior to enrolment.) |
| History of severe allergic reactions to vaccines or vaccine components including anaphylaxis and related symptoms such as hives, respiratory difficulty, angioedema, and/or abdominal pain |
| Immunoglobulin received within 60 days of first vaccine |
| Diagnosed autoimmune disease or immunodeficiency |
| Untreated or incompletely treated syphilis infection |
| Any clinically significant medical condition that would impact immune response, be a contraindication to repeated injections or blood draws, require active treatment or monitoring during follow-up period to prevent grave danger to the participant’s health, or have signs or symptoms that could be confused for a vaccine reaction |
| Any medical, psychiatric, occupational, or other condition that, in the judgment of the investigator, would interfere with, or serve as a contraindication to, protocol adherence, assessment of safety or reactogenicity, or a volunteer’s ability to give informed consent |
| Psychiatric condition that precludes compliance with the protocol. Specifically excluded are persons with psychoses within the past 3 years, ongoing risk for suicide, or history of suicide attempt or gesture within the past 3 years. |
| Current anti-tuberculosis treatment for active or latent tuberculosis |
| Asthma other than well controlled, mild asthma (daily use of recue inhaler, daily use of moderate/high dose inhaled steroids, >1 exacerbation in the last year requiring oral/parenteral steroids, or any exacerbation requiring urgent or emergent care would be excluded) |
| Any history of diabetes other than gestational diabetes |
| History of thyroidectomy or thyroid disease requiring medication within the last year |
| Hypertension that is not well controlled (systolic BP consistently < 140, diastolic BP consistently < 90) or any systolic BP > 150 or diastolic BP > 100 |
| Any bleeding disorder diagnosed by a doctor |
| Malignancy other than a fully excised malignancy deemed to have reasonable assurance of a sustained cure |
| Any seizure disorder requiring medication to treat or prevent seizures within the past 3 years, or any seizures within the past 3 years |
| History of asplenia (functional, surgical, or congenital) |
| History of hereditary angioedema, acquired angioedema, or idiopathic angioedema |

**Supplemental Table 3.** Details of the BAMA, ICS, ADCP and ADCC antigens, including HIV-1 viral strain information

| **Assay** | **Antigen/virus label used in plot/text** | **Antigen/virus class** | **Full antigen/virus name** | **Viral strain information:**  **Subtype.Country.Year.Stage*** |
| --- | --- | --- | --- | --- |
| BAMA | gp70_B.CaseA_V1_V2 | V1V2 | gp70_B.CaseA_V1_V2 | B.US.88.6 |
|  | gp70-TV1.GSKvacV1V2 | V1V2 | gp70TV1.GSKvacV1V2/293F | C.ZA.98.6 |
|  | 96ZM651.gp140C | gp140 | 96ZM651.gp140C.avi | C.ZM.96.6 |
|  | 1086C_D7gp120 | gp120 | 1086C_D7gp120.avi/293F | C.MW.04.1-2 |
|  | C.1086 V1/V2 | V1V2 | C.1086_V1_V2 Tags | C.MW.04.1-2 |
|  | 00MSA gp140 | gp140 | 00MSA 4076 gp140 |  |
|  | A1.con.env03 140 CF | gp140 | A1.con.env03 140 CF |  |
|  | Con 6 gp120/B | gp120 | Con 6 gp120/B | [Group M Consensus] |
|  | Con S gp140 CFI | gp140 | Con S gp140 CFI | [Group M Consensus] |
|  | TV1c8_D11gp120.avi/293F | gp120 | TV1c8_D11gp120.avi/293F | C.ZA.98.6 |
| ICS | 1086.C gp120 | gp120 | Env.1086.C | C.MW.04.1-2 |
|  | Env-ZM96 gp140 | gp140 | Env-ZM96.C | C.ZM.96.6 |
| ADCC-GTL | 1086C | gp120 | HIV 1086c_D7gp120.avi/293F | C 1086.c |
|  | TV1.C | gp120 | TV1c8_D11gp120.avi/293F | C TV-1 |
| ADCC-Luc | C Ce1086.c | Envelope-Infectious Molecular Clone | Ce1086_B2.LucR.T2A.ecto/293T/17 | C Ce1086.c |
|  | C TV-1 | Envelope-Infectious Molecular Clone | TV1.21.LucR.T2A.ecto/293T/17 | C TV-1 |
| *Subtype is denoted by a capital letter; country of origin is denoted by the 2 digit International Organization for Standardization code; year isolated is denoted by 2 digits; when country of origin and year isolated are unknown, they are denoted as “xx”; and stage is denoted by “a” (acute, if Fiebig stage is unknown) or “1”, “2”, “3”, “4”, “5”, or “6” (acute or early chronic, where the number or range corresponds to the Fiebig stage or range of stages when known).  **PSV = Env-pseudotyped virus | | | | |

**Supplemental Table 4.** Panel of antibodies used for Intracellular cytokine staining (ICS)

|  | **Specificity** | **Fluorochrome** | **Clone** | **Manufacturer** | **Catalogue number** |
| --- | --- | --- | --- | --- | --- |
| **Viability marker** | AViD | NA | NA | Life Technologies | L34957 |
| **Cellular surface markers** | CCR7 | BV785 | G043H7 | BioLegend | 353229 |
|  | CD14 | BV510* | M5E2 | BioLegend | 301842 |
|  | CD56 | BV570 | HCD56 | BioLegend | 318330 |
|  | CD45RA | APC H7 | HI100 | BD Biosciences | 560674 |
|  | CXCR5 | PE-Dazzle594 | J252D4 | BioLegend | 356928 |
|  | ICOS (CD278) | BV711 | DX29 | BD Biosciences | 563833 |
|  | PD-1 (CD279) | BV605 | EH12.2H7 | BioLegend | 329924 |
| **Intracellular markers** | CD3 | BUV737 | UCHT1 | BD Biosciences | 564307 |
|  | CD4 | BUV395 | SK3 | BD Biosciences | 563550 |
|  | CD8 | BV650 | RPA-T8 | BD Biosciences | 563821 |
|  | CD154 | APC | *TRAP-1* | BD Biosciences | 555702 |
|  | IFNγ | V450 | B27 | BD Biosciences | 560371 |
|  | Granzyme B | Alx700 | GB11 | BD Biosciences | 560213 |
|  | IL-2 | PE | *MQ1-17H12* | BD Biosciences | 559334 |
|  | IL-4 | PerCP-Cy5.5 | MP4-25D2 | BioLegend | 500822 |
|  | IL-17a | PE-Cy7 | *BL168* | BioLegend | 512315 |
|  | TNFα | FITC | MAb11 | eBioscience | 11-7349-82 |
| *CD14 and AViD are detected in the same channel | | | | | |

**Supplementary Table 5.** Severe (Grade 3) and potentially life-threatening (Grade 4) reactogenicity events

|  | **Placebo** | **T1** | **T2** | **T3** | **T4** | **T5** | **T6** | **T7** |
| --- | --- | --- | --- | --- | --- | --- | --- | --- |
| **Grade 3 Events** | | | | | | | | |
| Erythema | 0 | 0 | 4 | 2 | 0 | 2 | 3 | 3 |
| Induration | 0 | 0 | 2 | 0 | 0 | 1 | 2 | 2 |
| Tenderness | 0 | 0 | 0 | 0 | 0 | 1 | 0 | 0 |
| Fever | 0 | 0 | 0 | 1 | 0 | 2 | 1 | 0 |
| **Grade 4 Events** | | | | | | | | |
| Fever | 0 | 0 | 0 | 0 | 1 | 0 | 0 | 2 |

**Supplementary Table 6.** Related adverse events

| **MedDRA Preferred Term** | **Placebo** | **T1** | **T2** | **T3** | **T4** | **T5** | **T6** | **T7** | **Total** |
| --- | --- | --- | --- | --- | --- | --- | --- | --- | --- |
| **MILD** | | | | | | | | |  |
| Injection site pruritus | 0 | 0 | 1 | 0 | 5 | 3 | 0 | 1 | 10 |
| Injection site warmth | 0 | 0 | 0 | 0 | 0 | 1 | 0 | 0 | 1 |
| Pruritus | 0 | 0 | 0 | 0 | 0 | 1 | 0 | 0 | 1 |
| Lymph node pain | 0 | 1 | 0 | 0 | 0 | 1 | 0 | 0 | 2 |
| Lymphadenopathy | 0 | 0 | 0 | 0 | 0 | 0 | 0 | 1 | 1 |
| Abdominal pain upper | 0 | 0 | 0 | 0 | 0 | 0 | 1 | 0 | 1 |
| Diarrhoea | 0 | 1 | 0 | 0 | 0 | 0 | 0 | 0 | 1 |
| Dizziness | 0 | 1 | 0 | 1 | 0 | 0 | 0 | 0 | 2 |
| Pharyngitis | 0 | 0 | 0 | 0 | 0 | 0 | 1 | 0 | 1 |
| Alanine aminotransferase increased | 1 | 0 | 0 | 0 | 0 | 0 | 0 | 0 | 1 |
| Neutrophil count decreased | 0 | 2 | 0 | 0 | 0 | 0 | 0 | 0 | 2 |
| Decreased appetite | 0 | 1 | 0 | 0 | 0 | 0 | 0 | 0 | 1 |
| Pain in extremity | 0 | 0 | 0 | 0 | 0 | 0 | 2 | 0 | 2 |
| Dyspnoea | 0 | 0 | 0 | 0 | 0 | 0 | 0 | 1 | 1 |
| Hot flush | 0 | 0 | 0 | 0 | 0 | 0 | 1 | 0 | 1 |
| **TOTAL** | 1 | 5 | 1 | 1 | 4 | 6 | 4 | 3 | 28 |
| **MODERATE** | | | | | | | | |  |
| Axillary pain | 0 | 0 | 1 | 0 | 0 | 0 | 0 | 0 | 1 |
| Night sweats | 0 | 0 | 0 | 0 | 0 | 0 | 1 | 1 | 2 |
| Pruritus | 0 | 0 | 0 | 0 | 1 | 0 | 0 | 0 | 1 |
| **TOTAL** | 0 | 0 | 1 | 0 | 1 | 0 | 1 | 1 | 4 |

**
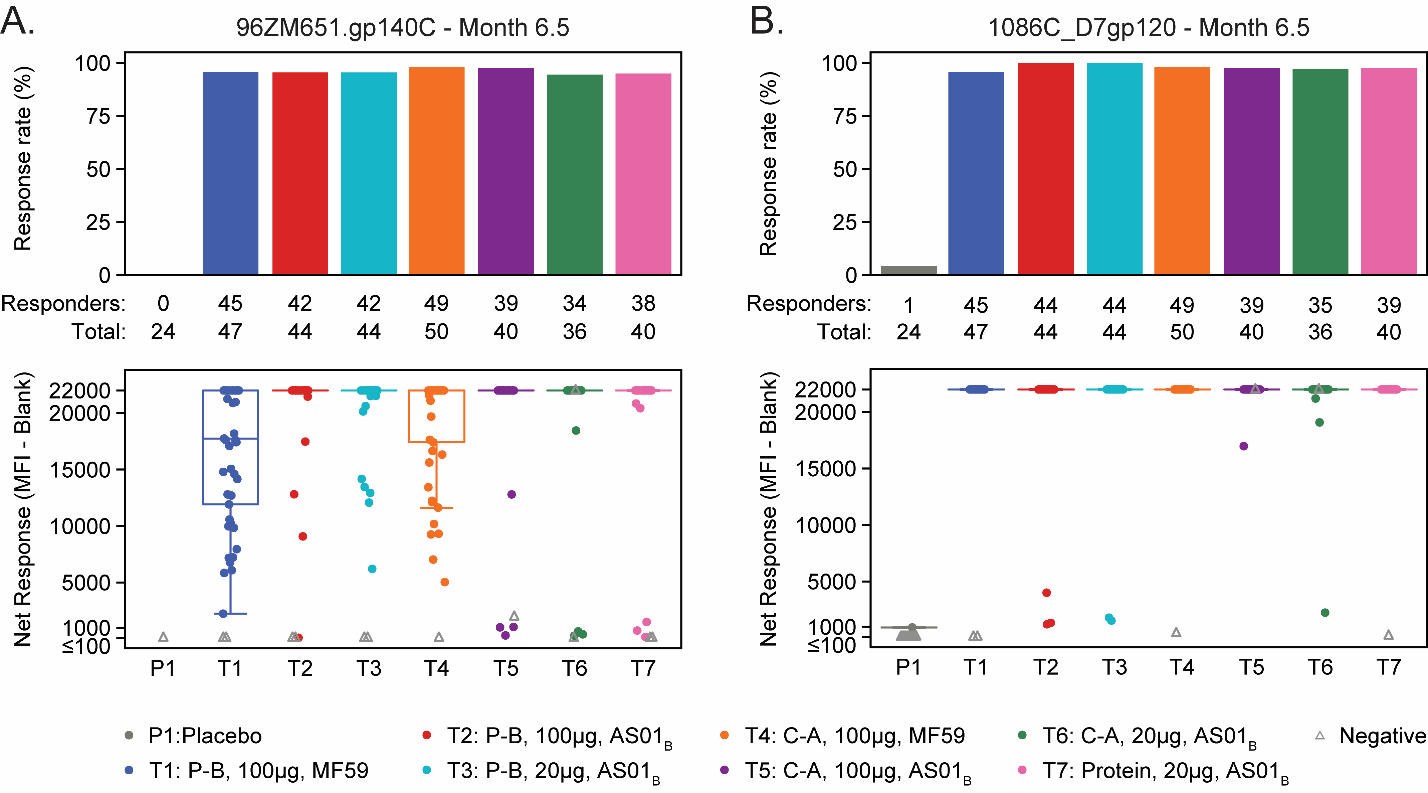
**

**Supplementary Figure 1.** IgG response rates (bar charts) and magnitude (boxplots) against 96ZM651.gp140C (A) and 1086C_D7gp120 (B) two weeks after the final immunization (Month 6.5) by treatment arm.


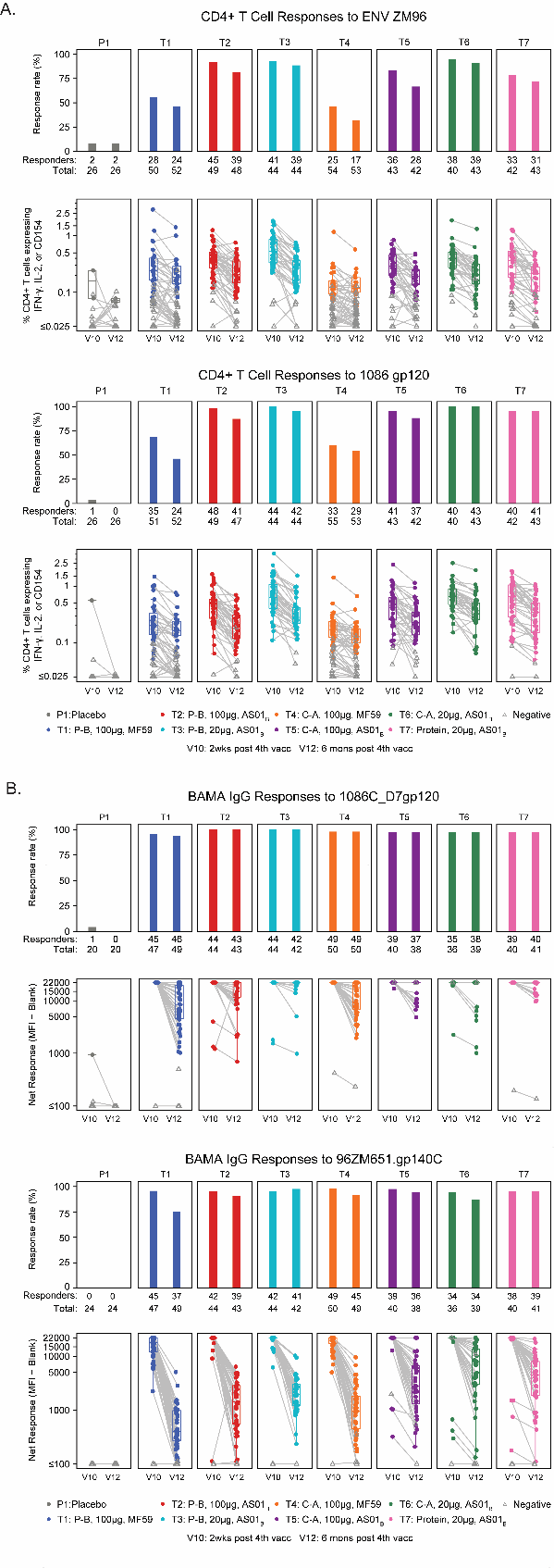


**Supplementary Figure 2.** Cellular responses 2 weeks and 6 months after completion of the primary vaccine regimen. Response rate (bar charts) and magnitude (boxplots) by treatment arm for the following vaccine-matched peptide pools: (A) CD4 T cell responses to Env-ZM96 gp140 and 1086 gp120 (B) IgG responses to 1086 gp120 and 96ZM. Bar charts show positive response rates. Boxplots show responses and are based on positive responders only (shown as colored circles), negative responders are shown as grey triangles.


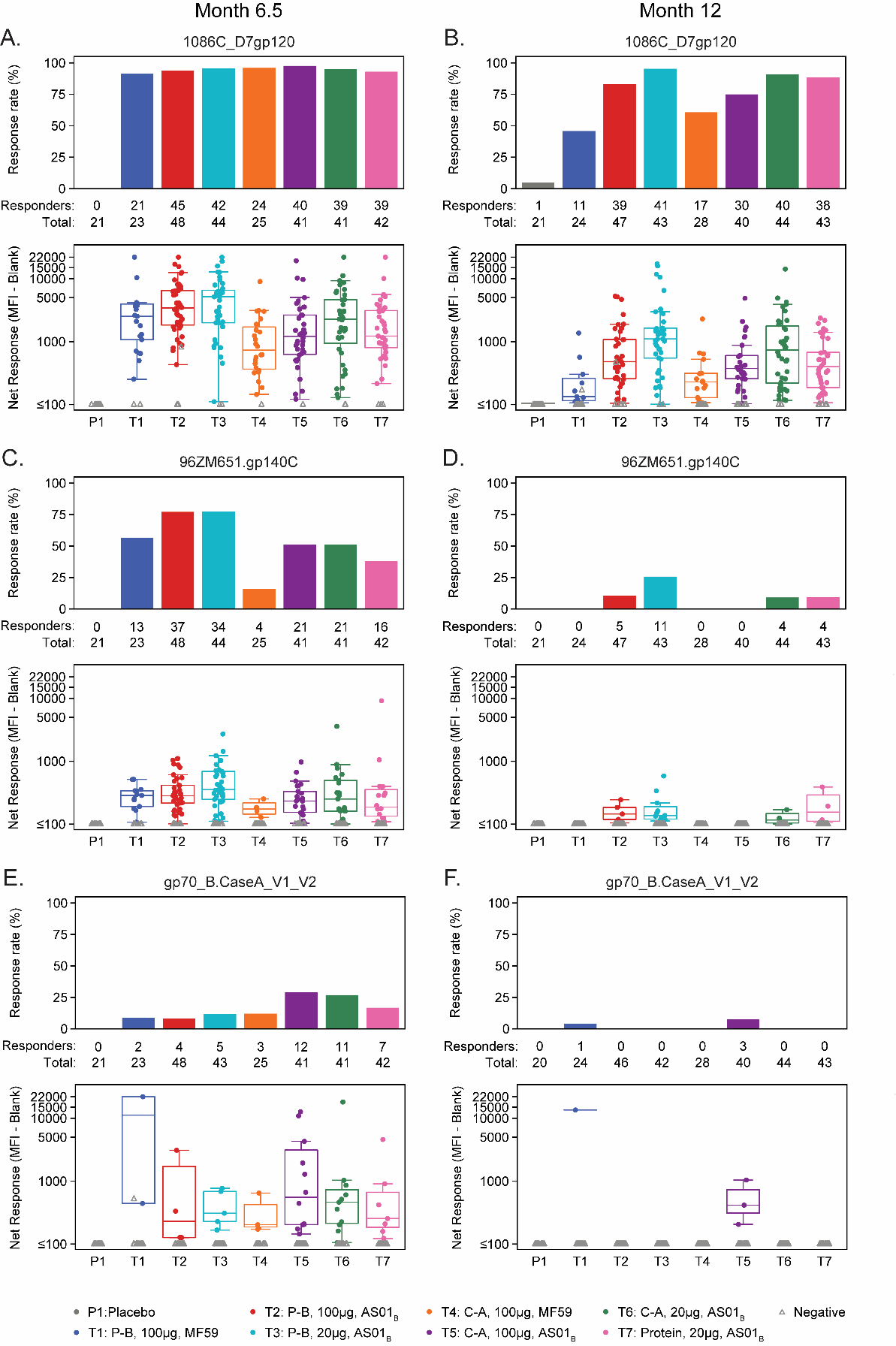
**S****upplementary Figure 3.** IgG3 responses 2 weeks (Month 6.5) and 6 months (Month 6.5) after completion of the primary vaccine regimen. IgG3 response rate (bar charts) and magnitude (boxplots) after the final immunization by treatment arm for the following antigens: 1086 gp120 (A and B), 96ZM gp140 (C and D), and gp70 B.CaseA V1V2 (E and F), 2 weeks and 6 months after the final vaccination. (P-B = Prime-Boost, C-A = Co-Administration).


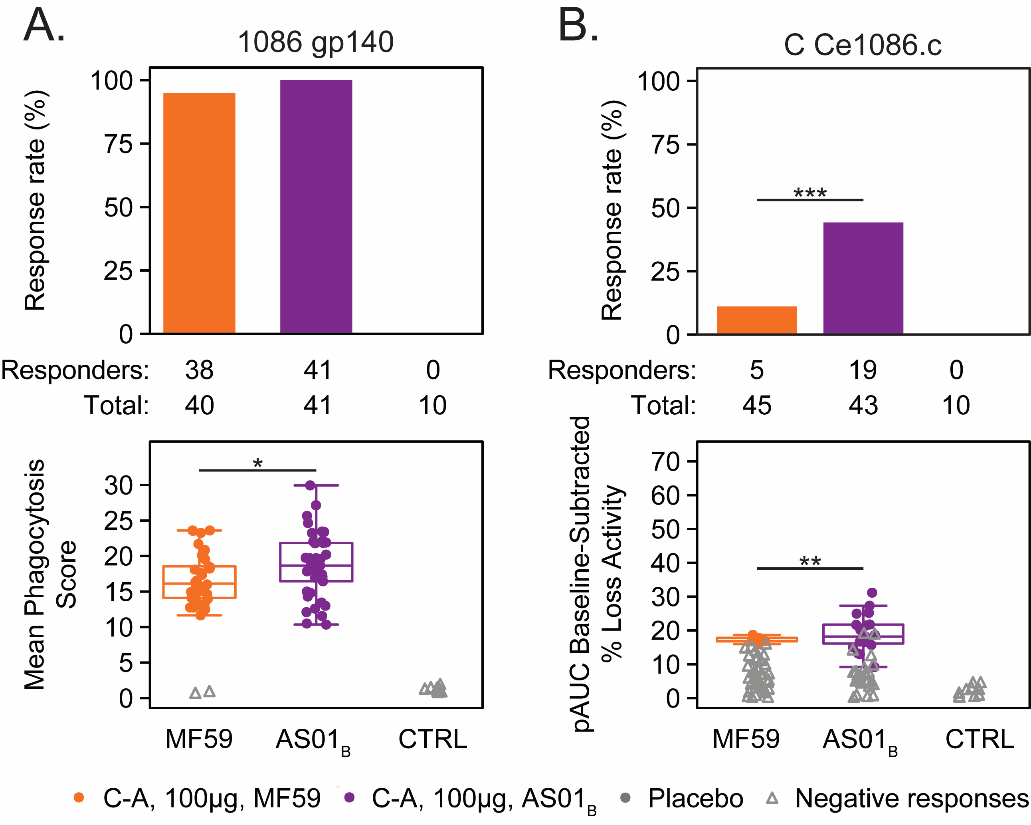


**Supplementary Figure 4.** Antibody-dependent cellular phagocytosis and cellular cytotoxicity 2 weeks after completion of the vaccine regimen. Antibody-dependent cellular phagocytosis scores to 1086 gp140 (A) and antibody-dependent cellular cytotoxicity scores to 1086C (B). Bar charts show positive response rates. Boxplots show responses and are based on positive responders only (shown as colored circles), negative responders are shown as grey triangles. *P<0.05, **P<0.01, ***P<0.001. (C-A = Co-Administration).


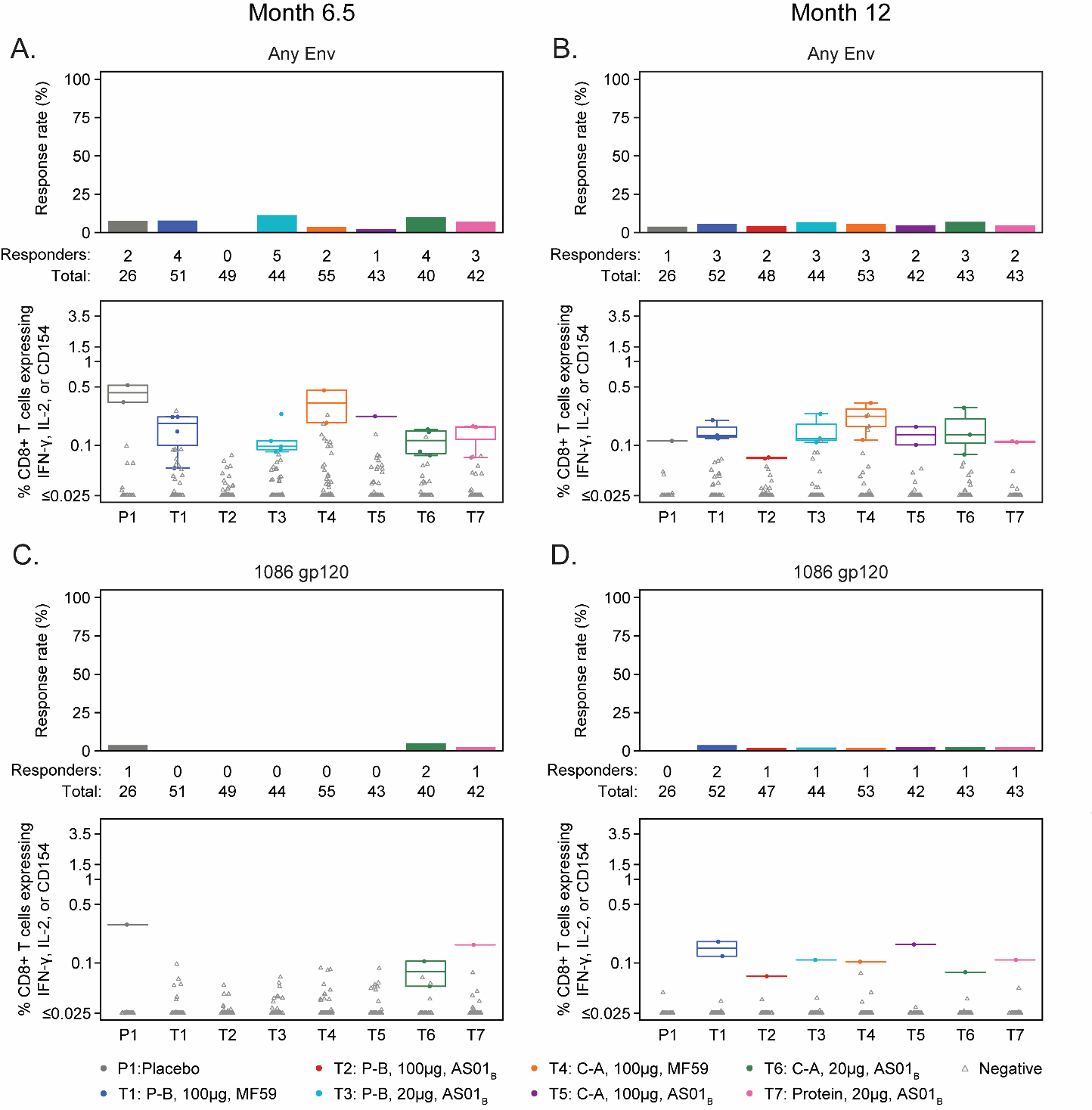
**Supplementary Figure 5.** The CD8+ T-cell responses 2 weeks (month 6.5) and 6 months (month 12) after completion of the primary vaccine regimen. Response rate (bar charts) and magnitude (boxplots) two weeks after and 6 months after the final immunization by treatment arm for the following vaccine-matched antigens (A and B) Any Env (the highest response to the Env peptide pools) (C and D) 1086 gp120. Bar charts show positive response rates. Boxplots show responses and are based on positive responders only (shown as colored circles), negative responders are shown as grey triangles.

**Supplementary Material References**

1. Pollara J, Bonsignori M, Moody MA, et al. HIV-1 vaccine-induced C1 and V2 Env-specific antibodies synergize for increased antiviral activities. *J Virol*. Jul 2014;88(14):7715-26. doi:10.1128/JVI.00156-14

2. Trkola A, Matthews J, Gordon C, Ketas T, Moore JP. A cell line-based neutralization assay for primary human immunodeficiency virus type 1 isolates that use either the CCR5 or the CXCR4 coreceptor. *J Virol*. Nov 199­­­9;73(11):8966-74. doi:10.1128/JVI.73.11.8966-8974.1999

3. Bull M, Lee D, Stucky J, et al. Defining blood processing parameters for optimal detection of cryopreserved antigen-specific responses for HIV vaccine trials. *J Immunol Methods*. Apr 30 2007;322(1-2):57-69. doi:10.1016/j.jim.2007.02.003

4. De Rosa SC, Carter DK, McElrath MJ. OMIP-014: validated multifunctional characterization of antigen-specific human T cells by intracellular cytokine staining. *Cytometry A*. Dec 2012;81(12):1019-21. doi:10.1002/cyto.a.22218

5. Hosseinipour MC, Innes C, Naidoo S, et al. Phase 1 Human Immunodeficiency Virus (HIV) Vaccine Trial to Evaluate the Safety and Immunogenicity of HIV Subtype C DNA and MF59-Adjuvanted Subtype C Envelope Protein. *Clin Infect Dis*. Jan 23 2021;72(1):50-60. doi:10.1093/cid/ciz1239
